# On the nature of photoluminescence in Bismuth-doped silica glass

**DOI:** 10.1038/s41598-017-03464-8

**Published:** 2017-06-09

**Authors:** Oleksii V. Laguta, Hicham El Hamzaoui, Mohamed Bouazaoui, Vladimir B. Arion, Igor M. Razdobreev

**Affiliations:** 1 Univ-Lille, CNRS, UMR 8523 – PhLAM - Physique des Lasers Atomes et Molécules, CERLA, F-59000 Lille, France; 2 Institute of Inorganic Chemistry, University of Vienna, Wahringer Str. 42, Vienna, A-1090 Austria

## Abstract

We report on the investigation of Bismuth-doped pure silica glass without other co-dopant by the tech- nique of magnetic circular dichroism (MCD), which allows the direct probing of the ground state of optical centres. Taking into account the results of conventional optical spectroscopy, we show that the observed MCD bands belong to the centre responsible for the red photoluminescence in this material. Measurements of the temperature and field dependences indicate that the MCD effect is caused by the even-electron system. This, however, opposes the widespread opinion that Bi^2+^ ions are the origin of red photoluminescence in Bismuth-doped silica glasses. On the other hand, the lasing centre responsi- ble for the near infrared photoluminescence does not exhibit any magnetic optical activity connected to its ground state. As a consequence, we conclude that the ground state of lasing centre is a magnetic singlet with the effective spin *S* = 0.

## Introduction

Bismuth-doped glasses are the subject of great research interest due to their potential applications for the development of new fiber amplifiers and tunable laser sources. Indeed, some Bismuth-doped glasses, depending on their compositions, show remarkably wide near infrared (NIR) photoluminescence (PL) bands in the spectral range 1100–1800 nm, covering all optical telecommunication windows where the traditional rare-earths-based glasses have a rather narrow operating spectral range^1–4^. However, even though NIR emission has been observed in a variety of Bismuth-doped glasses, silica based^5^, germanate^6^, chalcogenide^7^, fluoride^8^, and chloride^9^, the exact origin of this luminescence is still unknown. Because the identification of the nature of this NIR PL is crucial for further development of efficient light sources, research efforts have been devoted to the elucidation of the nature of active NIR-luminescent centre and a number of hypotheses have been proposed to explain the origin of NIR PL in Bismuth-doped materials. A detailed overview can be found in the literature^10, 11^. Note that in spite of the significant number of experimental and theoretical results, there is no consensus among researchers regarding the nature of NIR PL in the various Bismuth-doped glasses. Furthermore, some experimental observations appear controversial^11^. Because the conventional optical spectroscopy not always allows to draw unambiguous conclusions, new advanced experiments are highly desirable to clarify the nature of the luminescent centres in Bismuth-doped glasses.

Recently^12^, we used magnetic circular polarization of luminescence (MCPL) to investigate Bismuth-doped silica glass without other co-dopant. This technique enables the study of excited states (ES). Our experiments have shown that the lasing ES is a magnetic multiplet (NIR PL band 1400 nm). Furthermore, it was demonstrated that the magnetic field (MF) and temperature dependences of the MCPL signal correspond to an isolated non-Kramers doublet of the even-electron system. This important finding completely excludes any odd-electron system as a possible origin of the NIR PL. Unfortunately, based on the MCPL data alone, we were unable to reveal the magnetic multiplicity of the ground state (GS) of lasing optical centre. On the other hand, the well known red emitting centre, which was identified in all anterior investigations as Bi^2+^ ion^3, 11, 13^, was not studied in our recent work^12^. Moreover, to the best our knowledge, experiments directly proving the correctness of the assigning of red PL to the Bi^2+^ ion have so far not been presented. On the contrary, the absence of the electron paramagnetic resonance (EPR) signal in Bismuth-doped silica glass is likely to contradict this assumption. In this regard, the goal of the present paper was to clarify the spin multiplicity of the GS of both centres, the lasing one (responsible for the NIR PL) and the red PL emitting centre, by the magnetic circular dichroism (MCD).

The MCD is based on the Zeeman effect and provides valuable information about splitting of the electronic states in magnetic and crystal (CF) fields. Unlike the MCPL technique, in the MCD experiments we measure not the luminescence polarization but the difference in absorption of the right (σ^+^) and left (σ^−^) circularly polarized light in the external magnetic field. In general, MCD contains physically and mathematically separable contributions of three terms: ${\mathscr{A}}$, ${\mathcal B}$ and ${\mathscr{C}}$ ^14, 15^. The most interesting paramagnetic ${\mathscr{C}}$-term is non-zero if the ground state of the optical centre is degenerate. This term is magnetic field and temperature dependent. The field-induced mixing of the closely lying states produces a ${\mathcal B}$-term which depends only on the MF magnitude. The third, diamagnetic ${\mathscr{A}}$-term, is due to the excited state degeneracy and it is temperature independent. Due to these MCD term properties, this technique allows to investigate the nature of the GS of paramagnetic centre by measuring the temperature and magnetic field dependences of the MCD signal.

In the present paper, for the first time, we investigate the Bismuth-doped silica glass without other co-dopant by the MCD technique in the wavelength range 300–1600 nm. We show that GS of the lasing centre is a magnetic singlet *S* = 0. This result allows us to conclude that the attribution of the NIR PL to Bi^+^ ion in this material is not contradictory. The second very interesting and somewhat unexpected result is that the GS of centre responsible for the red PL in this glass is not just a magnetic multiplet corresponding to the half-integer effective spin, but it is also split in a zero magnetic field. The very high energy of this splitting indicates that it is due to the crystal field. It turns out that this centre is also the even-electron system. Thus, we conclude that the assignment of the red PL in this material to Bi^2+^ ion that was made in all previous works was wrong. This means that the Bi^2+^ ions are simply absent in this material.

## Results and Discussion

**Figure 1:**
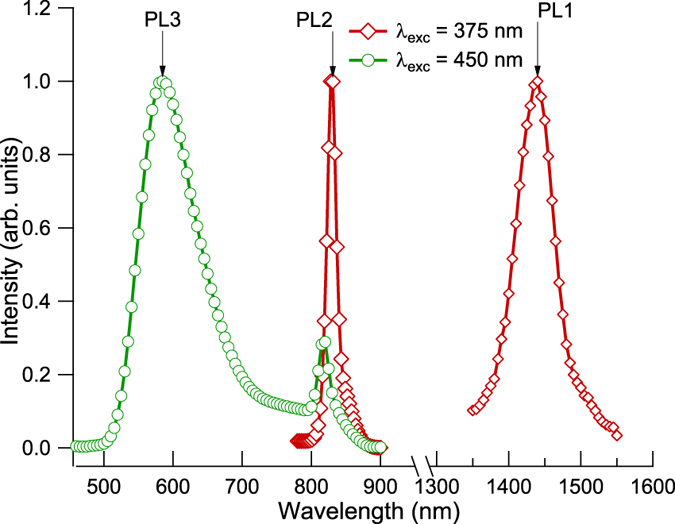
Spectra of luminescence in visible and NIR ranges under different excitation wavelengths.

**Figure 2:**
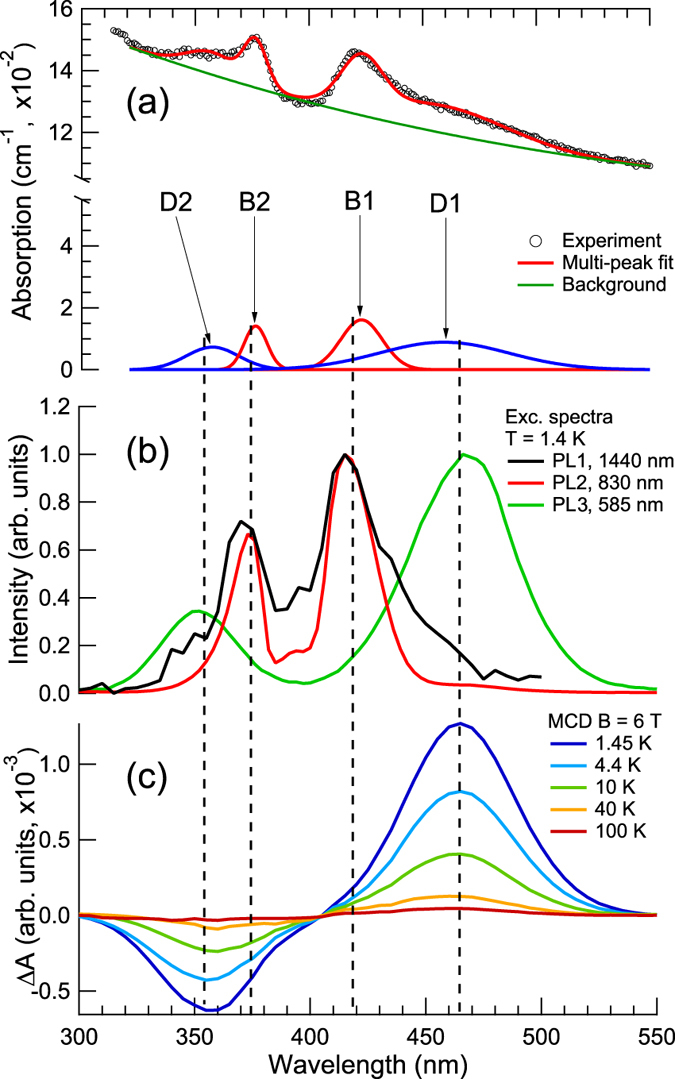
(**a**) Absorption spectrum recorded at room temperature. (**b**) Excitation spectra of PL1, PL2 and PL3 luminescence bands recorded at 1.4 K. (**c**) MCD spectra recorded at various temperatures in magnetic field B = 6 T.

In Fig. 1 we show the PL spectra recorded at 1.4 K in the visible and NIR spectral regions under excitation at 375 and 450 nm. Three intense PL bands, labeled as PL1, PL2, PL3 with peaks at 1440, 830 and 585 nm, are well known and correspond to those reported earlier^16^ (see also for the completeness)^3, 17^. It is worth noting that the narrow PL2 band can be observed under both excitation wavelengths, though it is much stronger and red-shifted under excitation at 375 nm, which can be explained by the inhomogeneous broadening and partial superposition of the absorption (excitation) spectra corresponding to different centres as it can be seen in Fig. 2(a,b).

In Fig. 2(a) we show the absorption spectrum, recorded in the range 300–550 nm. This spectrum consists of two complex bands with maxima near 380 and 420 nm. Each of these bands can be decomposed into two sub-bands as it is suggested by the Gaussian fit also shown in Fig. 2(a). Labels D1, B1, B2 and D2 correspond to bands with peaks at 460, 410, 375 and 350 nm, respectively. In Fig. 2(b) the PL excitation spectra recorded at 1440, 830 and 585 nm are shown. There are two bands in the excitation spectrum of the luminescence band PL2 recorded in the spectral region 300–550 nm with the intensity peaks at 415 and 375 nm. These bands can be put in the direct correspondence to the bands B1 and B2 in the Gaussian deconvolution of absorption spectrum. The excitation spectrum of the NIR PL1 band also consists of two bands centred at 370 and 420 nm. The excitation spectrum of PL3 band also reveals two bands with maxima at 465 and 350 nm. Obviously, they should be put in correspondence to the absorption bands D1 and D2. It is seen from comparison of the excitation spectra that the luminescence bands PL1 and PL2 belong to the same centre, while the band PL3 to another one.

The MCD spectra recorded at fixed MF 6 T and different temperatures in the spectral range 300–550 nm are shown in Fig. 2(c). They consist of two bands, one positive and one negative, whose position and spectral width are very close to the absorption and excitation bands D1 and D2, respectively. In the magnetic field **B** of 6 T at 1.45 K we measured Δ*A* = −0.63 × 10^−3^ and 1.27 × 10^−3^ at 350 and 465 nm, respectively. The MCD spectra are temperature dependent, which definitely indicates the spin multiplicity of the corresponding ground state. The MCD bands exhibit the same temperature dependence as expected for two different bands of the same centre. The absence of the MCD signal in B1 and B2 absorption bands indicates that the GS of corresponding centre is a magnetic singlet with effective spin *S* = 0. It should be also noted that we could not detect any MCD signal in the whole spectral range between 550 and 1600 nm. This is not surprising taking into account previous reports^3, 17, 18^ where it was shown that the NIR PL and absorption bands at 1440, 830, 375 (B1) and 415 nm (B2) belong to the same centre. This observation, in principle, does not contradict the assumption that the Bi^+^ ion is responsible for the NIR PL in this material^19^.

**Figure 3:**
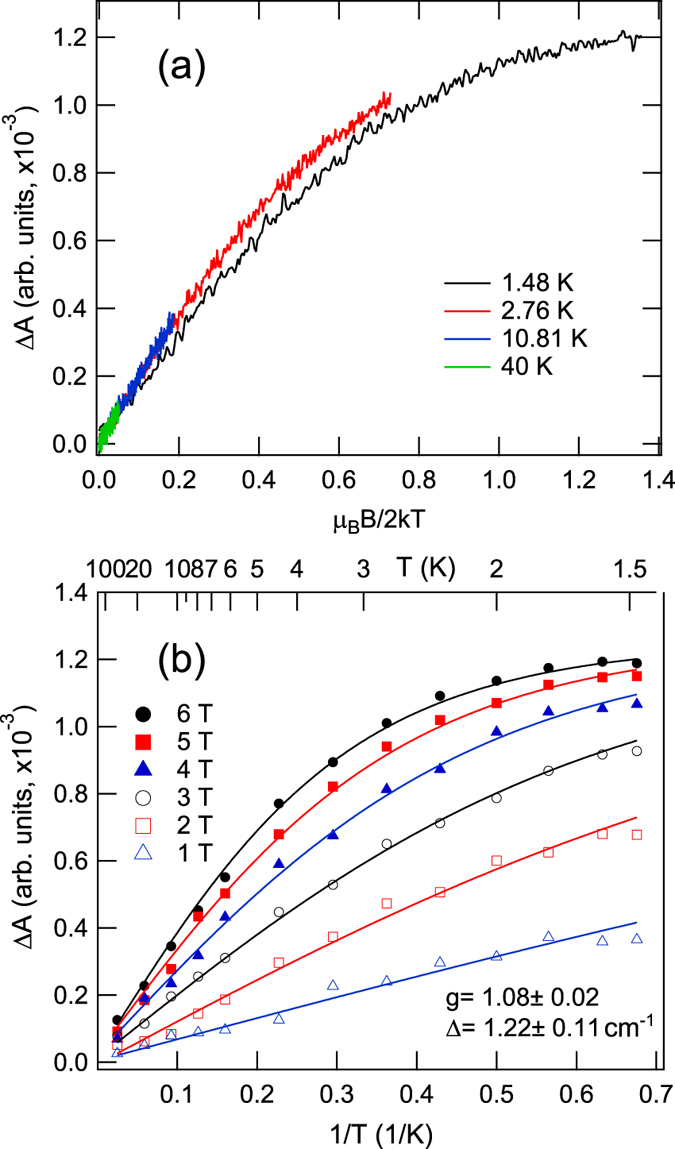
Magnetic field (**a**) and temperature (**b**) dependences of MCD at 465 nm (MCD and absorption band D1). Solid lines in (**b**) represent the fit by Eq. (2).

The most valuable information about the MCD active centre can be obtained from the analysis of saturation of magnetization. Magnetic field dependences of MCD were recorded at different fixed temperatures in two spectral points corresponding to the band peaks: 465 and 352 nm (bands D1 and D2, respectively). Because qualitatively two measured curve subsets were identical, in Fig. 3(a) we show only the one corresponding to the band D1. It is seen that the isotherms do not superimpose but exhibit a small degree of nesting. The curves tend towards the saturation at high MF and low temperature. Temperature dependences of MCD recorded at fixed magnitudes of the external magnetic field plotted as a function of 1/*T* are shown in Fig. 3(b). The MCD signal increases with decreasing temperature and it becomes saturated at high magnetic field and low temperature.

**Figure 4:**
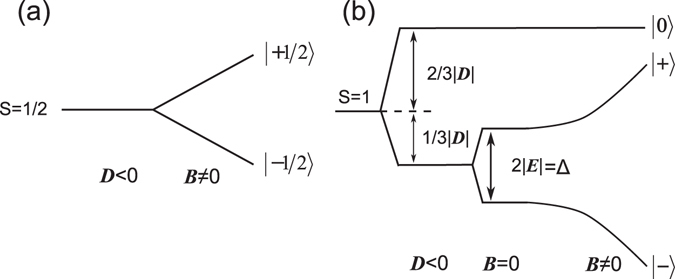
Energy level diagrams corresponding to (**a**) *S* = 1/2 and (**b**) *S* = 1 effective spin in the assumption of the Hamiltonian (1).

The analysis of saturation of magnetization data was performed in terms of the spin Hamiltonian with axial (*D*) and rhombic (*E*) components of the crystal field^20, 21^:

1 $$ {\mathcal H} =D\{{S}_{z}^{2}-\frac{1}{3}S(S+\mathrm{1)}\}+E({S}_{x}^{2}-{S}_{y}^{2})+{g}_{\parallel }{\mu }_{B}B{S}_{z}\,\cos \,\theta ,$$

where *S* is the effective spin, *θ* is the angle between the applied magnetic field *B* and the symmetry axis (z-axis), g_||_ is the axial component of g-tensor and *μ*_*B*_ is the Bohr magneton. In the frame of this model, the odd- and even-electron systems exhibit qualitatively different behaviour. In particular, the crystal field cannot remove completely the degeneracy in the case of a half-integer effective spin *S* = 1/2, 3/2, … (Kramers doublet), as it is shown in Fig. 4(a) for the simplest case of *S* = 1/2. Kramers doublet can be split only by the MF, and when it is applied parallel to z-axis (*θ* = 0), the gap between spin sub-levels increases linearly with MF. In the case of integer spin, the crystal field potential can completely remove the degeneracy of the state as it is shown in Fig. 4(b) for the system with *S* = 1. In order to reach the lowermost energy position for the non-Kramers doublet *M*_*s*_ = ±1, the axial component of the crystal field must be negative (*D* < 0). The rhombic component of the crystal field removes the residual degeneracy, producing the so-called zero-field splitting (ZFS) with corresponding energy gap between *M*_*s*_ = ±1 sub-levels Δ = 2|E|. MCD can be observed only in the presence of MF because of the peculiarities of |±〉 wave functions^21^.

Quantitative information about the studied system was obtained by fitting the saturation experimental data to the following equation^21, 22^:

2 $${\rm{\Delta }}A={A}^{sat}{\int }_{0}^{1}\frac{2g{\mu }_{B}B\,{\cos }^{2}\,\theta }{\sqrt{{{\rm{\Delta }}}^{2}+{\mathrm{(2}g{\mu }_{B}B\cos \theta )}^{2}}}\times \,\tanh (\frac{\sqrt{{{\rm{\Delta }}}^{2}+{\mathrm{(2}g{\mu }_{B}B\cos \theta )}^{2}}}{2kT})d(\cos \,\theta ),$$

where *A* ^*sat*^ is the saturation value of MCD and *g* is the effective *g*-factor, which depends on the value of the effective spin *S*. The simultaneous fit of saturation curves shown in Fig. 3(b) results in low *g*-value *g* *=* 1.08 ± 0.02 and Δ = 1.22 ± 0.11 cm^−1^ in the assumption that the GS is triplet, *S* = 1. The non-zero value of ZFS energy Δ definitely indicates that the optical centre responsible for the observed MCD in Bismuth-doped silica glass is a system with even number of electrons. As a result, any odd-electron system, and Bi^2+^ ions in particular, should be excluded as possible origin of the MCD bands. Moreover, the even-electron systems with a non-degenerate GS, like Bi^5+^ and Bi^+^ ions, also cannot cause the MCD. Amorphous silica glass has a very large family of the point defects^23, 24^. However, there are no suitable among the known defects, which would have a triplet ground state and the absorption bands in the range 300–600 nm. In this regard, we put forward the hypothesis that the MCD bands Di and red luminescence are related to the unknown defect in the glass network. Probably, Bismuth doping leads to the formation of some new kind of defect or it changes the properties of the existing one. We would also like to note that similar conclusions about the nature of the red luminescence were made by us in the detailed magneto-optical investigation of Bismuth-doped aluminosilicate glass^25^.

Our experiments show that the observation of the conventional EPR in Bismuth-doped silica glass is possible. However, attempts to detect with conventional spectrometers an EPR signal, that could be assigned to a Bismuth-related centre, failed^10^. The first and main reason for this is that all the EPR experiments, at the best of our knowledge, were performed with the microwave frequencies in the X- band (9.5 ± 0.5 GHz), which is too low to match measured here ${\rm{ZFS}}\simeq 36.6$ GHz. The second reason, but much less important, is that the microwave transitions $|-\rangle \iff |+\rangle$ are allowed in the configuration when the microwave MF is parallel to the static magnetic field **B**, whereas in the most conventional EPR spectrometers these fields are perpendicular to each other. Finally, in the glass materials all the paramagnetic centres are randomly oriented. This causes an extremely large broadening of the resonance line so that its intensity may be below the limit of sensitivity of the spectrometer.

## Conclusion

In conclusion, magnetic circular dichroism has been studied in a Bismuth-doped silica glass without other co-dopant. It was shown that the ground state of the optical centre responsible for the red photoluminescence is the spin multiplet. Magnetic field and temperature dependences of MCD revealed the zero-field splitting in the GS. This fact implies that the red luminescence is caused by the even-electron system. This interpretation, in turn, denies the assumption that the Bi^2+^ ions are the source of red luminescence in the studied material. Thus, the magneto-optical methods do not reveal any trace of the Bi^2+^ ion in the studied material. Also, our investigation made prediction of the experimental conditions for observing the conventional EPR signal. Finally, we conclude that the lasing centre responsible for the NIR PL has a non-degenerate GS (effective spin *S* = 0), that, in principle, does not contradict the hypothesis that this photoluminescence is caused by the electronic transitions in Bi^+^ ions.

### Materials and Methods

The investigated bulk SiO_2_:Bi samples with the dimensions 2 mm × 4 mm × 5 mm were similar to that reported by us previously^16^. The only difference was the enhanced atomic ratio Bi/Si, which was estimated ≈400 ppm using the electron probe microanalysis (EPMA).

**Figure 5:**
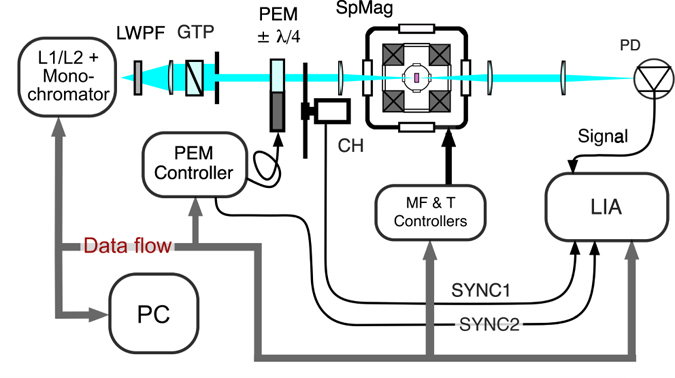
Setup for MCD measurements. L1/L2 - UV Xenon or quartz tungsten lamp, LWPF - long wave pass filter; GTP - Glan-Thompson polarizer; PEM - photoelastic modulator; CH - chopper; SpMag - cryomagnetic system; PD - Si or Ge amplified photo-detector; LIA - multi-frequency lock-in amplifier.

In Fig. 5 we show the setup of the MCD spectrometer. The sample was placed in a helium closed-cycle magneto-optical cryostat SpectromagPT (Oxford Instruments). The system provides magnetic field up to 7 T with the homogeneity of 0.01% over 10 mm diameter spherical volume and temperature stabilization in 1.4–300 K range. As a light source we used a quartz tungsten or UV enhanced Xenon lamp. Light from the source was dispersed by a monochromator with a spectral resolution 2.5 and 5 nm in the range 350–900 and 900–1600 nm, respectively. After the collimating lens and exchangeable long wave pass filter (LWPF) the light was linearly polarized with a large aperture Glan-Thompson polarizer. The photoelastic modulator (PEM, II/FS20 optical head, Hinds Instruments) acted as a dynamic wave plate oriented at 45° to the polarization plane of the incident light beam. In order to transform the linear polarization of light to the circular, σ^+^ or σ^−^, PEM periodically introduced the retardation $\delta ={\delta }_{0}\,\sin \,\mathrm{(2}\pi {f}_{PEM}t)$, where *δ*_0_ = *λ*/4 is the peak retardation, *f*_*PEM*_ = 20 KHz is the modulator frequency. Then the light beam was chopped at low frequency ${f}_{CH}\simeq 300$ Hz. After passing through the sample it was detected by the amplified photodetector (Si or Ge photodiode). In the case of a small dichroism Δ*A* < 0.1 the MCD signal is Δ*A* = *V*_*PEM*_/*V*_*CH*_, where *V*_*PEM*_ and *V*_*CH*_ are the signals at PEM’s and chopper’s frequencies, respectively. The phase-sensitive technique with a multi-frequency lock-in amplifier (MFLI, Zurich Instruments) was used to record *V*_*PEM*_ and *V*_*CH*_ signals simultaneously.

## References

[1] Bufetov I, Dianov E (2009). Bi-doped fiber lasers. Laser Phys. Lett..

[2] Dianov EM (2012). Bismuth-doped optical fibers: a challenging active medium for near-IR lasers and optical amplifiers. Light: Science & Applications.

[3] Bufetov I (2014). Bi-doped optical fibers and fiber lasers. IEEE J. Sel. Top. Quant..

[4] Firstov SV (2016). A 23-db bismuth-doped optical fiber amplifier for a 1700-nm band. Sci. Reports.

[5] Fujimoto Y, Nakatsuka M (2001). Infrared luminescence from bismuth-doped silica glass. Jpn. J. Appl. Phys..

[6] Peng M (2004). Bismuth- and aluminum-codoped germanium oxide glasses for super-broadband optical amplification. Opt. Lett..

[7] Dong G (2008). Broadband infrared luminescence from Bismuth-doped GeS_2_-Ga_2_S_3_ chalcogenide glasses. Chinese Phys. Lett..

[8] Romanov A (2011). Near-IR luminescence from subvalent bismuth species in fluoride glass. Opt. Mater..

[9] Romanov AN (2012). On the origin of near-IR luminescence in Bi-doped materials (II). Subvalent monocation Bi^+^ and cluster Bi_5_^3+^ luminescence in AlCl_3_/ZnCl_2_/BiCl_3_ chloride glass. Opt. Express.

[10] Peng M (2011). Discussion on the origin of NIR emission from Bi-doped materials. J. Non-Cryst. Solids.

[11] Sun H-T, Zhou J, Qiu J (2014). Recent advances in bismuth activated photonic materials. Progress in Materials Science.

[12] Laguta O, El Hamzaoui H, Bouazaoui M, Arion V, Razdobreev I (2015). Magnetic circular polarization of luminescence in bismuth-doped silica glass. Optica.

[13] Dianov EM (2015). Nature of Bi-related near IR active centers in glasses: state of the art and first reliable results. Laser Phys. Lett..

[14] Serber R (1932). The theory of the Faraday effect in molecules. Phys. Rev.

[15] Stephens P (1974). Magnetic circular dichroism. Annu. Rev. Phys. Chem..

[16] Razdobreev I (2010). Optical spectroscopy of Bismuth-doped pure silica fiber preform. Opt. Lett..

[17] Firstov SV (2011). Combined excitation-emission spectroscopy of bismuth active centers in optical fibers. Opt. Express.

[18] Firstov SV (2013). Anti-Stokes luminescence in Bismuth-doped silica and germania-based fibers. Opt. Express.

[19] Romanov A, Haula E, Shashkin D, Vtyurina D, Korchak V (2017). On the origin of near-IR luminescence in SiO_2_ glass with bismuth as the single dopant. Formation of the photoluminescent univalent bismuth silanolate by SiO_2_ surface modification. J. Lumin..

[20] Abragam, A. & Bleany, B. *Electron Paramagnetic Resonance of Transition Ions* (Oxford University Press, 1970).

[21] Solomon EI, Pavel E, Loeb K, Campochiaro C (1995). Magnetic circular dichroism spectroscopy as a probe of the geometric and electronic structure of non-heme ferrous enzymes. Coord. Chem. Rev..

[22] Laguta O, Denker B, Galagan B, Sverchkov S, Razdobreev I (2016). Magnetic optical activity in Bi-doped Mg-Al-Si glass. Opt. Quant. Electron..

[23] Skuja L (1998). Optically active oxygen-deficiency-related centers in amorphous silicon dioxide. J. Non-Cryst. Solids.

[24] Pantelides S (2008). The E’ center and oxygen vacancies in SiO_2_. J. Non-Cryst. Solids.

[25] Laguta A, Denker B, Sverchkov S, Razdobreev I (2017). Magneto-optical studies of bismuth-doped MgO-Al_2_O_3_-SiO_2_ glass. On the nature of infrared luminescence. Quantum Electron..

